# A Closer Look at Penetration: The Efficacy Gap in Vitamin C Products

**DOI:** 10.1111/jocd.70851

**Published:** 2026-04-03

**Authors:** McKenzie E. Maloney, May Hall, Tatiana Kononov, Alisar Zahr

**Affiliations:** ^1^ Department of Internal Medicine Massachusetts General Hospital Boston Massachusetts USA; ^2^ Advanced Dermatology and Skin Surgery Asheville North Carolina USA; ^3^ Revision Skincare Irving Texas USA

**Abbreviations**
AA: ascorbic acid
ROS: reactive oxygen species
SA: sodium ascorbate
THD: tetrahexyldecyl
UVB: ultraviolet light B
Vit C: vitamin C

Oxidative stress drives cutaneous aging through free radicals, specifically reactive oxygen species (ROS). ROS production is triggered by both internal and external pathways. For example, ultraviolet light B (UVB) irradiation generates ROS, causing cellular dysfunction, DNA damage, and carcinogenesis [1]. To counteract oxidative stress, a complex system of enzymatic and nonenzymatic antioxidants synergistically quenches ROS and repairs cellular damage [2]. As skin ages, levels of nonenzymatic antioxidants and enzymatic antioxidant activity decrease [3]. Therefore, to replenish inherent nonenzymatic antioxidation levels, the topical application of nonenzymatic antioxidants, such as vitamins, has emerged as a promising adjuvant.

Vitamin C (Vit C) is a popular antioxidant formulated into cosmeceutical products to mitigate oxidative stress, reduce melanin synthesis, and enhance collagen production [4]. However, because of its potent hydrophilic properties, developing a stable product with sufficient penetration into the skin while protecting the skin barrier remains challenging. As a result, several derivatives of Vit C are utilized in marketed cosmeceutical products. While there is ample data evaluating their clinical efficacy, a dearth of literature compares their penetration [5]. Therefore, this study compared the combined epidermal and dermal penetration of three Vit C derivatives over 72 h.

A Strat‐M Membrane (Millipore, Germany) was used to investigate the intraepidermal/intradermal penetration of marketed Vit C cosmeceutical products. This synthetic membrane was developed to mimic human skin, composed of a hydrophobic stratum corneum and an underlying fibrous layer resembling the dermis, and is a validated method for comparative absorption analysis [6]. Compared to animal models, such as porcine skin, Strat‐M Membrane's absorption more closely resembles human skin [6]. For this comparative study, three marketed cosmeceutical products were tested using 50 μL of 30% tetrahexyldecyl (THD) ascorbate emulsion, 15% L‐ascorbic acid (AA) serum, and encapsulated sodium ascorbate (SA); the absorption was measured with High Performance Chromatography (HPLC).

After 6 h, the penetration was 6%, 4.8%, and 0.225% for THD ascorbate, AA, and SA, respectively. After 72 h, the penetration was 14%, 0.36%, and 0.36% for THD ascorbate, AA, and SA, respectively (Figure 1). THD ascorbate had 38 times greater absorption compared to AA and SA. These results highlight the superiority of THD ascorbate at penetrating the epidermis and dermis compared to the other marketed Vit C cosmeceutical products.

**FIGURE 1:**
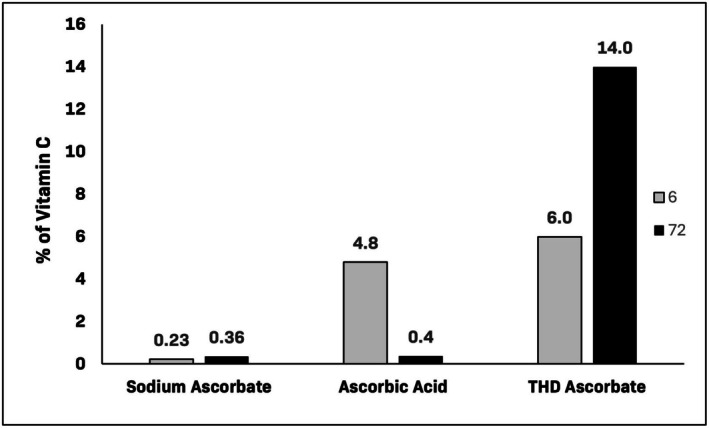
Dermal absorption of vitamin C formulations after 6 and 72 h using a StratM Membrane.

With photoaging, the natural level of Vit C in skin decreased by 31% compared to young skin (mean age 23 vs. 76 years) [3]. This reduction in antioxidant potential is further compounded by decreased antioxidant enzymatic activity, shifting the skin into oxidative stress and accelerating the aging process [2, 3]. Therefore, replenishing antioxidants in the intracellular environment is essential for revitalizing inherent antioxidant pathways, returning oxidative balance to the microenvironment, and diminishing the aging process.

The functionality of topical Vit C derivatives relies on both their absorptive capacity and stability. The absorptive capacity or penetration reflects the derivative's ability to mimic the skin's natural lipophilic composition. This study highlighted the superiority of THD ascorbate, a lipophilic derivative, and the inefficiency of AA and SA, hydrophilic derivatives, in penetrating the dermis. Secondly, the stability of the formulation is essential to prevent its premature oxidation. For example, SA readily converts to AA and sodium hydroxide in aqueous solution. While AA is a potent antioxidant, its instability in aqueous solution predisposes it to rapid oxidation and degradation. The combined effects of penetration and stability determine intracellular concentrations of vitamin C, which is critical, as some of its functions, such as neocollagenesis, are concentration‐dependent [5]. Derivatives with enhanced stability and absorption more efficiently increase intracellular vitamin C concentrations and trigger the biostimulatory response. Therefore, SA and AA are undesirable formulations for cosmetical products compared to THD ascorbate, which has excellent absorption, stability, and clinical results.

## Funding

This work was sponsored by Revision Skincare LLC.

## Consent

The authors have nothing to report.

## Conflicts of Interest

McKenzie E. Maloney and Tatiana Kononov are consultants at Revision Skincare. May Hall is a board‐certified dermatologist and key opinion leader in vitamin C technology. Alisar Zahr is a full‐time Revision Skincare employee.

## Data Availability

The data that support the findings of this study are available on request from the corresponding author. The data are not publicly available due to privacy or ethical restrictions.
